# Modified carbon paste ion selective electrode for determining Cr(iii) ions in aqueous solutions and some real samples using tetragonal zirconia nanoparticles†

**DOI:** 10.1039/d3ra01563g

**Published:** 2023-04-11

**Authors:** Omar A. Fouad, Mohamed M. S. Wahsh, Gehad G. Mohamed, Maher M. I. El Dessouky, Maysa R. Mostafa

**Affiliations:** a Chemistry Department, Faculty of Science, Cairo University 12613 Giza Egypt oahmed@sci.cu.edu.eg; b Refractories, Ceramics and Building Materials Department, National Research Centre 12622 Cairo Egypt; c Nanoscience Department, Basic and Applied Sciences Institute, Egypt-Japan University of Science and Technology New Borg El Arab Alexandria 21934 Egypt

## Abstract

Tetragonal zirconia (t-ZrO_2_) nanoparticles (ionophore) are used in newly designed and improved ion selective electrodes for chromium ion detection as an alternative, low-cost, high-precision, and selectivity method. Tetragonal zirconia nanoparticles were synthesized using a modified co-precipitation technique and calcined at 1000 °C for an hour. The phase composition, surface area, microstructure, pore size and particle size of synthesized t-ZrO_2_ nanoparticles were examined using the X-ray diffraction (XRD), Brunauer–Emmett–Teller (BET), transmission electron microscope (TEM) and scanning electron microscopy (SEM) attached with an EDAX unit, respectively. Results from XRD showed that the t-zirconia was synthesized and have nanocrystallites size about 20.2 nm. The nano size of t-ZrO_2_ was confirmed by the SEM and TEM (the particle size between 26.48 and 40.4 nm), the mesoporous character (average pore size about 4.868 nm) and large surface area (76.2802 m^2^ g^−1^) was confirmed by BET analysis. The paste composition with 67.3 : 30.5 : 2.7 (wt%) graphite, t-ZrO_2_, and TCP, respectively, exhibited the best results. With a detection limit of 1.0 × 10^−8^ mol L^−1^, the electrode displayed a good Nernstian slope of 19.50 ± 0.10 mV decade^−1^ over the concentration range from 1.0 × 10^−2^ to 1.0 × 10^−8^ mol L^−1^ of Cr(iii) ions. The built-in sensor displayed a quick response time (7 s), was highly thermally stable in the range of 10 to 60 °C without departing from Nernstian behaviour and could be used for about 60 days in the pH range of 2.0 to 6.0. The electrode demonstrated excellent selectivity for the Cr(iii) ion towards a variety of metal ions. For chromium ion determination, numerous spiked real samples, including honey, water, tea, coffee, milk, cheese, and cosmetics, were used. Validation methods were used, and the results showed that there is no significant difference between the two methods (ICP and ISE) at a 95% confidence level. In several real water samples, the estimated limits of detection, limits of quantification, percent recovery, standard deviation, and relative standard deviation showed the effectiveness of the proposed electrode in the potentiometric detection of Cr(iii) ions.

## Abstract 2

Tetragonal zirconia (t-ZrO_2_) nanoparticles (ionophore) are used in newly designed and improved ion selective electrodes for chromium ion detection as an alternative, low-cost, high-precision, and selectivity method.

## Graphical abstract

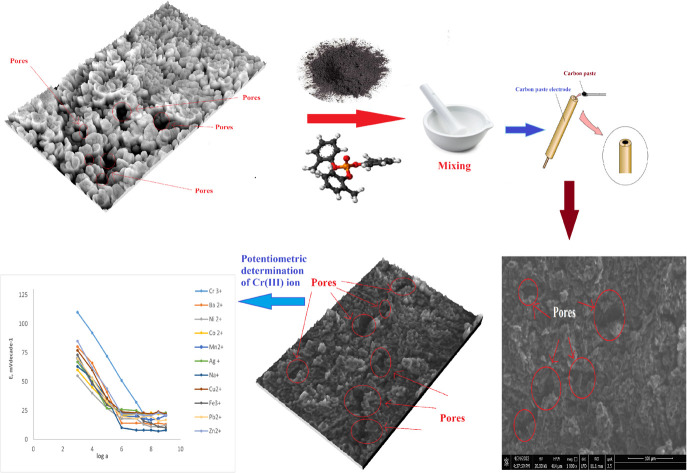

## 1. Introduction

Chromium is applied to synthesize a variety of alloys like stainless steel and is also used to harden steel. Additionally, plastics can be chrome plated. Most leather is tanned with chromium. Chromium compounds are utilized as industrial catalysts and pigments (green, yellow, red, and orange colors). It also serves an important biological function by assisting in the utilization of glucose, a nutrient needed by all cells,^1^ it also performs a crucial metabolic function. In water-based solutions, the established oxidation states of chromium in aqueous solutions are Cr(iii) and Cr(vi). Compared to Cr(vi) compounds, Cr(iii) is thought to be less hazardous.^2–4^ But when taken in excess, about 1 mg daily, it is harmful.^5,6^

Alternative techniques, particularly spectrophotometry,^7^ isotope dilution mass spectrometry,^8^ cloud point extraction and flame atomic absorption spectrometry,^9^ HPLC,^10^ chromatography^10^ inductively coupled plasma mass spectrometry,^11^ have been considered for the monitoring of Cr(iii) ions. Even though these techniques produced accurate, selective, and sensitive results, they are hindered by expensive, specialized gear besides laborious and time-consuming sample preparation. However, electrochemical techniques, which are safe for the environment, have the benefits of high sensitivity, simplicity of use, and mobility.^4,12^ Ion concentrations in various substances can now be directly determined potentiometrically using ion-selective electrodes (ISEs).^4,13–16^

One of the extremely important ISE is modified carbon paste-based sensors (MCPEs) which formed from blending of pasting liquid (plasticizer), conductive graphite powder and ion carrier and offer a lot of merits include being simple to apply to colored and turbid liquids, affordable, providing satisfactory selectivity, having a minimal detection limit, possessing outstanding accuracy, and offering a wide concentration range. The ion carrier employed in the MCPE determines all of these benefits.^17–19^ Due to their great repeatability, ability to be altered by a span of modifiers, and usage of less expensive materials, they have been favored as sensing materials and are frequently employed for voltametric measurements.^20,21^

Nanoparticles exhibiting conduction sites for electron transfer, for instance oxide NPs, semiconductor NPs, metal NPs, and even composite NPs, are extensively utilized in electrochemical sensors.^22^ One of the most significant nanoparticles is zirconium oxide, sometimes denoted to as zirconia, is a white, crystalline solid utilized in coatings, ceramics, electronics, pigments, and some medical applications. It has exceptional mechanical characteristics and is very resistant to mechanical stress and cracking. ZrO_2_ was well recognized for having a great resistance to heat as well as a thermal expansion coefficient of 1.08 × 10^−5^ K^−1^ and a melting point of 2700 °C. Because it doesn't absorb neutrons, is superconductive at low temperatures, and be able to employed to create superconducting magnets, zirconium is ideal for use in nuclear power plants.^23^ It is non-reactive and chemically inert.^24^

At standard pressure and ambient temperature (STP), pure zirconia occurs as a monoclinic (m-ZrO_2_) phase that changes into a tetragonal (t-ZrO_2_) phase at about 1170 °C, a cubic fluorite structure (c-ZrO_2_) at about 2370 °C.^25,26^ The structure of pure ZrO_2_ materials is significantly destroyed during the transition between the three ZrO_2_ crystal forms, which is often associated by volume changes and shear strain.^26,27^ Pure ZrO_2_ is easily transformed throughout a heating–cooling cycle from the t-ZrO_2_ phase to m-ZrO_2_ phase, resulting in a volume shift that is primarily represented by a 5% volume expansion during cooling and a 3.25% volume shrinkage during heating.^28^ Due to the breaking of its products and the buildup of tensile stress throughout pure ZrO_2_ material, which generates a toughening effect, this significantly restricts the usage of pure ZrO_2_ materials. As a result, pure ZrO_2_ must be stabilized to generate the tetragonal and/or cubic phases, which give material has outstanding mechanical, thermal, and electrical properties.^25,26,28^

In an effort to control the structure of ZrO_2_ materials and stop this t → m-ZrO_2_ transformation, cations having a radii larger than those of Zr(iv) are incorporated into ZrO_2_ lattices to replace part of the Zr(iv) lattice sites. At the same time, alternative solid solutions are developed in these ZrO_2_ materials *via* systematic doping, that preserves the steady phase structure of the consequent doped ZrO_2_ materials at ambient temperature and delivers a toughening impact for pure ZrO_2_ materials.^26,29^ Usually, the main components of doped stabilizers are alkaline earth and rare earth oxides. Ionic oxides and Zr(iv) should have radii that differ from one another by no more than 40%.^28,30^ Especially noteworthy are the more often used MgO,^31^ CaO,^32^ Y_2_O_3_,^28,33^ and CeO_2_.^28,34^ As a consequence of combining with these oxides, the m-ZrO_2_ phase at ambient temperature prefers highly symmetric metastable structures (c and t-ZrO_2_) that are comparable to those in pure zirconia but with dopant ions replaced on Zr(iv) positions and with a portion of oxygen positions unoccupied to preserve charge balance.^35,36^ The crystal of ZrO_2_ may be either fully or partially stabilized; however, fully stabilized zirconia (FSZ) (c-ZrO_2_) has less advantageous mechanical properties than partially stabilized zirconia (PSZ), therefore the stabilizer concentrations should be lower than those required for full stabilization of ZrO_2_.

Magnesium partially stabilized zirconia (Mg-PSZ) reveals high *R*-curve conduct owing to transference toughening, with a peak fracture toughness >20 MPam^1/2^.^37,38^ The stress-induced t → m-phase conversion is the fundamental mechanism. Through utilizing eutectoid and/or sub-eutectoid ageing process, along with an appropriate cooling rate, it is feasible to increase the quantity of reconfigurable tetragonal phase in Mg-PSZ and achieved desired fracture toughness.^37,38^

This work's objectives are to detail the synthesis of nano-t-ZrO_2_, characterize it, integrate it within CPEs as a modifier, and measure its performance features for Cr(iii) ion detection. Several characterization methods, such as powder X-ray diffraction (XRD), scanning electron microscopy (SEM) with EDAX, Transmission electron microscope (TEM) and Brunauer–Emmett–Teller (BET) analysis, were exploited to acquire comprehension of the structure and behaviors of the nano t-ZrO_2_. For MCPE the impacts of t-ZrO_2_-NPs concentration, plasticizer, pH, temperature, selectivity, and longevity were comprehensively evaluated. Both pure Cr(iii) and several authentic samples could be detected using the MCPE as potentiometric sensors. The criteria for approach validity were looked at.

## 2. Experimental

### 2.1. Materials and methods

In every experiment, analytical reagent grade chemicals and reagents were employed, along with double distilled water. Sigma-Aldrich Chemie GmbH provided the zirconyl chloride octahydrate (purity 98%) and magnesium chloride hexahydrate (purity 99%) used in this study. Ammonia solution came from Riedel-deHaen in Germany, and Acros Organics in the USA provided the chromium chloride hexahydrate. Whereas dioctyl phthalate (DOP) and dibutyl phthalate (DBP) were provided by BHD, and *o*-nitrophenyloctylether (*o*-NPOE) was supplied by Fluka. Aldrich provided the tricresylphosphate (TCP) and graphite powder (1–2 μm). Ascorbic acid, CoCl_2_·6H_2_O, PbNO_3_, NiCl_2_·6H_2_O, AgNO_3_, NaCl, CuCl_2_·2H_2_O, MnCl_2_·4H_2_O and varied metals chlorides like Fe(iii), Mg(ii), Ca(ii), Co(ii) and Ba(ii) obtained from El-Nasr Company (Egypt) were utilized as interfering materials in the analytical category. All apparatus used are discussed in our previous studies.^4,12^

### 2.2. Synthesis of t-ZrO_2_ nanoparticles

Using a modified co-precipitation process, the t-ZrO_2_ nanoparticles were created. Using distilled water as a solvent, zirconyl chloride octahydrate solution (1 M) was created. The zirconyl chloride octahydrate solutions were then mixed with the magnesium chloride hexahydrate solutions in a quantity, corresponding to MgO weight percentage of 14. This ratio has been chosen to be consistent with the earlier studies that indicate pure t-ZrO_2_ is present when the weight percentage of MgO is equal to 14, and less than this ratio the polyphase (monoclinic, tetragonal and cubic phases) ZrO_2_ is frequently detected.^39^ To stimulate a homogenous solution, the prepared solutions were blended with steady stirring for one hour. The Zr(iv) and Mg(ii) chloride solutions were treated with ammonia dropwise until the pH was 10.5. This resulted in the preparation of Zr(iv) and Mg(ii) hydroxides. The metal ions solutions were filtered through ashless filter paper (Whatman® Grade 41, England) to separate the precipitates, and the chloride ions were then eliminated by washing the mixture three times with distilled water. The precipitates have been initially dried for twenty-four hours at 90 °C, then calcined for 1 hour at 1000 °C with a 5 °C min^−1^ heating rate. Characterization of the synthesized pure t-ZrO_2_ NPs have been acquired using X-ray diffraction, a scanning electron microscope (SEM) connected to an EDAX unit, transmission electron microscope (TEM) and Brunauer–Emmett–Teller (BET) analysis.

### 2.3. MCPE preparation

The black paste is homogeneously ground with 0.250 g, (5, 10, 15) mg, and 0.12 g of graphite powder, t-ZrO_2_-NPs, and plasticizer, respectively, which are mixed in a mortar and pestle. The mixture was gently stuffed into the electrode's tube over one side then left there for 24 hours in distilled water to obstruct air intrusion. A fine tissue was utilized to sweep the graphite paste's surface prior to the test.

Calibration was carried out by measuring the potential of^.^1.0 × 10^−10^–1.0 × 10^−2^ mol L^−1^ of Cr(iii) solution starting from low to high concentration by transferring 5 mL aliquots of Cr(iii) into 25 mL beakers at 25 °C (pH = 4) followed by immersing the CPE in conjunction with Ag/AgCl electrode in the solution. The potential change was plotted against the logarithm of Cr(iii) ion.

### 2.4. Preparation of samples for the analysis

#### 2.4.1. Milk and cheese samples

The cheese sample was processed handmade cheese that is commonly offered in local markets and is fashioned by adding vinegar to milk after boiling. A sample of milk was imported from an Egyptian market. After adding 50.0 mL of each specimen in 2 duplicates to an uncovered porcelain crucible that had previously been pretreated with 1 mL of strong nitric acid (65%), the crucible subsequently progressively heated over a hot plate (IKA, Tbilisi, Georgia) till fume terminated. For the cheeses, 10.0 g of every sample was inserted into two duplicate uncoated porcelain crucibles rinsed with 1 mL of pure nitric acid (65%) blended by deionized water (1 : 1) then left for fifteen min. Following that, both specimens have been ashed in a muffle furnace (Nabertherm, L15/11, Germany) that had been preheated to 250 °C and had been elevated in temperature progressively (by 50 °C after 30 min.) to 450 °C. Ashing was accomplished till grey ash was collected, the leftover subsequently dissolved in mild nitric acid, and the combination was progressively dried at 140 °C on a hot plate (IKA, Staufen, Germany). Samples were placed back into the muffle furnace after cooling for 30 minutes at 300 °C. Up till white ash was acquired, the stage before it was repeated. Finally, 5 mL of strong nitric acid was used to dissolve the ash samples, and that solution was then diluted to 25 mL (50 mL diluted for cheese samples). Two blank samples were performed using deionized water and filters in the same manner as when exploiting the reagents alone.^40^

#### 2.4.2. Red tea sample

Five grams of tea were utilized to generate heating samples, which were subsequently filtered and completed in 50 mL of deionized water. The resultant solutions' pH was adjusted to be 5.^41^ Mate tea was acquired from a Korean market, whereas red tea was purchased from an Egyptian store.

#### 2.4.3. Coffee sample

Roasted and crushed coffee were placed in boiling water (95 to 100 °C), filtered, and then utilized to generate the coffee infuse. Afterward, 25 mL of the infuse that had been made by volume was condensed to produce around 2.5 mL of the final volume in a greenhouse with good ventilation and air interchange (Tecnal brand, TE-394/2 model, Piracicaba, SP, Brazil) at 60 °C. By soaking the crushed coffee sample with a 3 : 1 ratio of nitric and perchloric acids, the sample was mineralized.^42^

#### 2.4.4. Cosmetics eye shadow sample

Each sample received 10 mL of digested acid (3 : 1 v/v HCl : HNO_3_) after being pipetted into a digestion test tube with care at a weight of 0.5 g each sample. This was set for 30 minutes on the hot plate. Following digestion, the samples were brought up to a volume of 50 mL with distilled water, allowed to cool to ambient temperature, and then filtered.^43^

#### 2.4.5. Honey sample

Each honey sample was weighed into 24 separate digestion tubes, each holding about 1 g. Then, using a measuring cylinder, about 10 mL of aqua regia were inserted to the digestion tubes containing the honey samples. To create a homogenous mixture, the aforementioned combination was gently shaken. The tubes holding the samples comprising aqua regia and honey were subsequently placed with digestion blocks on a hot plate. The setup hot plate's temperature was set to 100 °C. When the thermometer reached a temperature of 100 °C, the digestive tubes were put into the blocks. Sample solutions were heated for an hour, throughout which time they turned from deep yellow to pale yellow. Sample solutions that had been digested were then allowed to reach room temperature. Finally, a Whatman filter paper has been exploited to penetrate the solution. Distilled water was append to the digested sample solutions until they reached a final volume of 50 mL.^44^

## 3. Outcomes and interpretation

### 3.1. XRD for the prepared nano-t-ZrO_2_

**Fig. 1:**
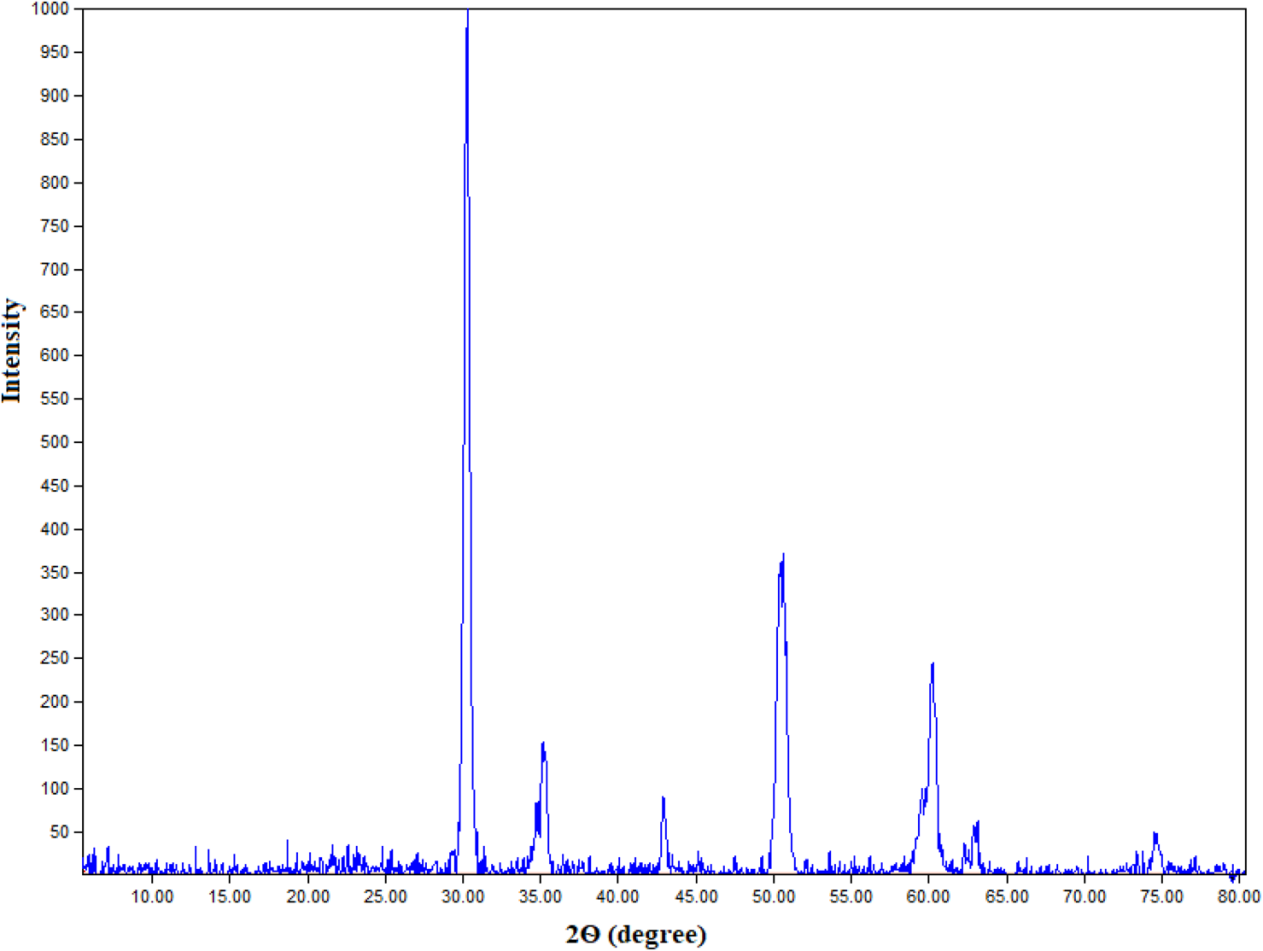
XRD patterns of the t-ZrO_2_ nanoparticles.

Fig. 1 exposes the XRD patterns of the prepared nano t-ZrO_2_. Without any diffraction peaks corresponding to other phases, t-ZrO_2_ is the only significant phase that has been identified. According to the XRD pattern of pure t-ZrO_2_, the sharp diffraction peaks at 30.27°, 35.22°, 42.92°, 50.50°, 60.25°, 62.97°, and 74.6° were attributed to the (0 1 1), (1 1 2), (0 1 1), (1 2 1), (0 2 2), and (2 2 0) planes, respectively. The *P*4_2_/*nmc* (137) space group, card number 2300612, and the tetragonal structure all suit these sharp peaks. So, in light of these XRD results, the pure t-ZrO_2_ was successfully synthesized using 14 weight percent of MgO as a stabilizer. Additionally, the XRD pattern's existence of significant sharp peaks reveals that the synthesized nano t-ZrO_2_ was crystalline and nanometric in scale. The average crystallite size of prepared nanoparticles is 20.2 nm (derived from the Debye–Scherrer equation).^4,45^

### 3.2. SEM in conjunction with EDAX of t-ZrO_2_

**Fig. 2:**
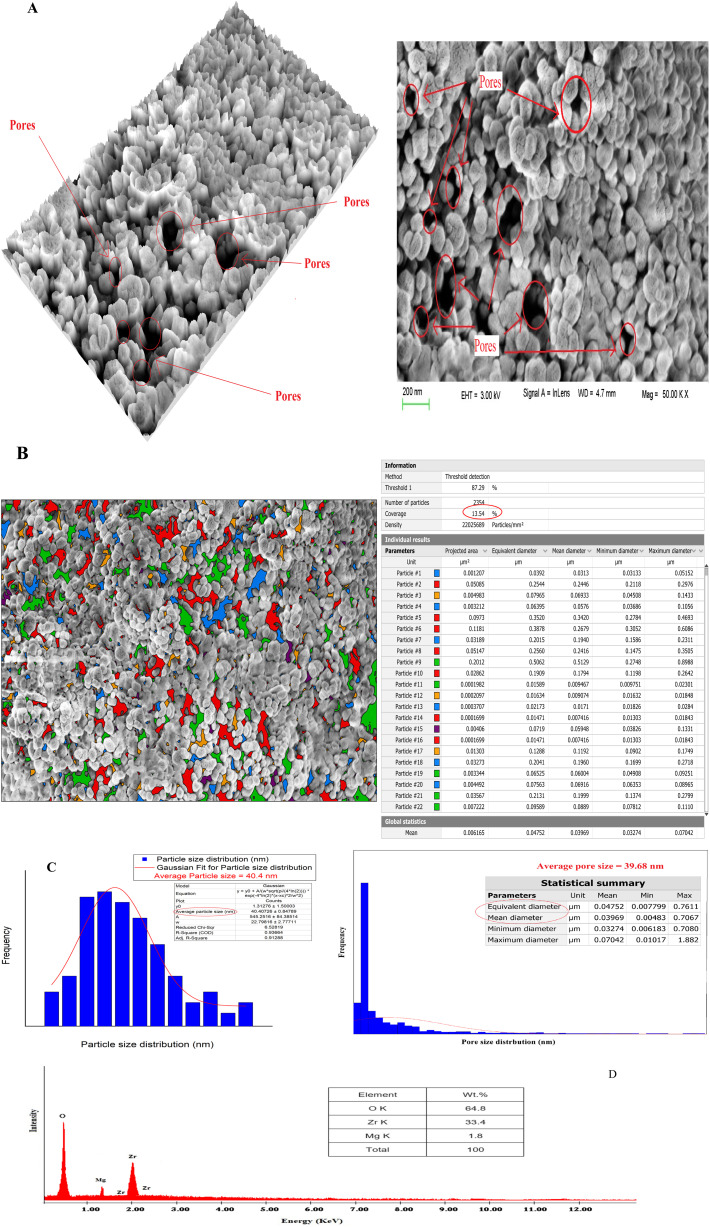
The SEM microphotographs (A), particle and pore size distributions (B and C) and EDAX data (D) of the prepared t-ZrO_2_ nanoparticles.

The shape and microstructure of the surface of the prepared t-ZrO_2_ nanoparticles were examined using scanning electron microscopy (SEM) in conjunction with EDAX, Digital Surf's Mountains Lab®, and Java 1.8.0172 with ImageJ (1.53e) software.^46,47^ SEM images of the t-ZrO_2_ nanoparticles showed their spherical-like shape, homogeneous matrix, smooth surface, and porous structure (Fig. (2A)). The SEM microphotographs also revealed the presence of different pore sizes and particles on the synthesized t-ZrO_2_ nanoparticles.

The pores in the sample were seen to take on different colors using the color threshold method on SEM microphotographs with a 1 μm scale, as discovered in Fig. (2B). Using Digital Surf's Mountains Lab® program, it was determined that the overall percentage of pores and the average size of the pores were 13.54% and 39.68 nm, respectively.

Additionally, the Java 1.8.0172 with ImageJ (1.53e) tool was established to produce the Gaussian mixture model and histogram in Fig. (2C) to estimate the distribution of particle sizes. The average particle size of 40.4 nm was found for the t-ZrO_2_ nanoparticles. The chemical composition of t-ZrO_2_ nanoparticles was identified using EDAX data (Fig. (2D)). Magnesium constitutes about 1.8% of the composition, followed by zirconium (33.4%) and oxygen (64.8%). The EDAX dates exposed that the synthesized t-ZrO_2_ nanoparticles sample had a homogenous distribution of all its constituent parts.

### 3.3. Transmission electron microscopy (TEM) of nano t-zirconia

**Fig. 3:**
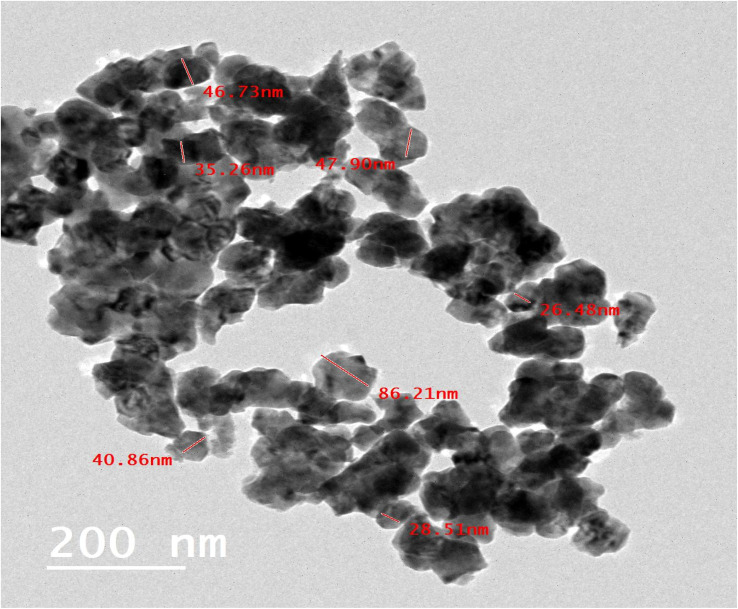
The high-resolution TEM image (HR-TEM) of the prepared t-ZrO_2_ nanoparticles.

The TEM was used to examine the morphology and particle sizes of the synthesized t-ZrO_2_ nanoparticles. Fig. (3) demonstrates that the average particle size of the t-ZrO_2_ NPs falls between 26.48 nm and 86.21 nm, which is in good consistency with the average crystallite size determined from XRD data (20.2 nm) and the average particle size determined from the SEM image analysis (40.4 nm). The outcome also supports the nanoscale synthesis of t-ZrO_2_.

### 3.4. BET-analysis

The nitrogen adsorption (BET) measurements for t-ZrO_2_ nanoparticles revealed a high surface area about 76.2802 m^2^ g^−1^ with an average pore size about 4.868 nm and pore volume about 0.186 cm^3^ g^−1^ which verified that the mesoporous structure of the nano t-ZrO_2_.^12^

### 3.5. Electrochemical determination of Cr(iii) ion with utilized electrodes

Many modified CPEs with nano t-zirconia as a powerful electroactive material, graphite sheets as a matrices and TCP as a solvent moderator have been synthesized to establish the best paste composition with the best effectiveness that complies to the Nernst equation.^16^ In (ESI† Table S1) the outcomes are displayed. The calibration was performed by submerging the electrodes associated with the double junction Ag/AgCl reference electrode in Cr(iii) ion solutions with concentrations ranging from 1.0 × 10^−8^ to 1.0 × 10^−2^ mol L^−1^ which covers a wider range of concentrations than that reported in Barati *et al.*,^48^ in which CPEs were evaluated throughout the interval from 1.0 × 10^−3^ to 1.0 × 10^−6^ mol L^−1^.

Different modified CPEs were established with varied nano t-ZrO_2_ contents, such as 5, 10, and 15 mg. Additionally, the effects of various plasticizers, such as DHP, DBP, TCP, DOP, and NPOE, were investigated. ESI† Tables (S1 and S2) pointed out the fact that the modified CPEs that had been treated with 10 mg of t-ZrO_2_ and plasticized with TCP had the best Nernstian slope of 19.5 ± 0.10 mV decade^−1^. The high *R*-square (0.998) is another evidence of the significance of the suggested model and the accuracy fit the model.

### 3.6. Surface analysis of the MCPE

**Fig. 4:**
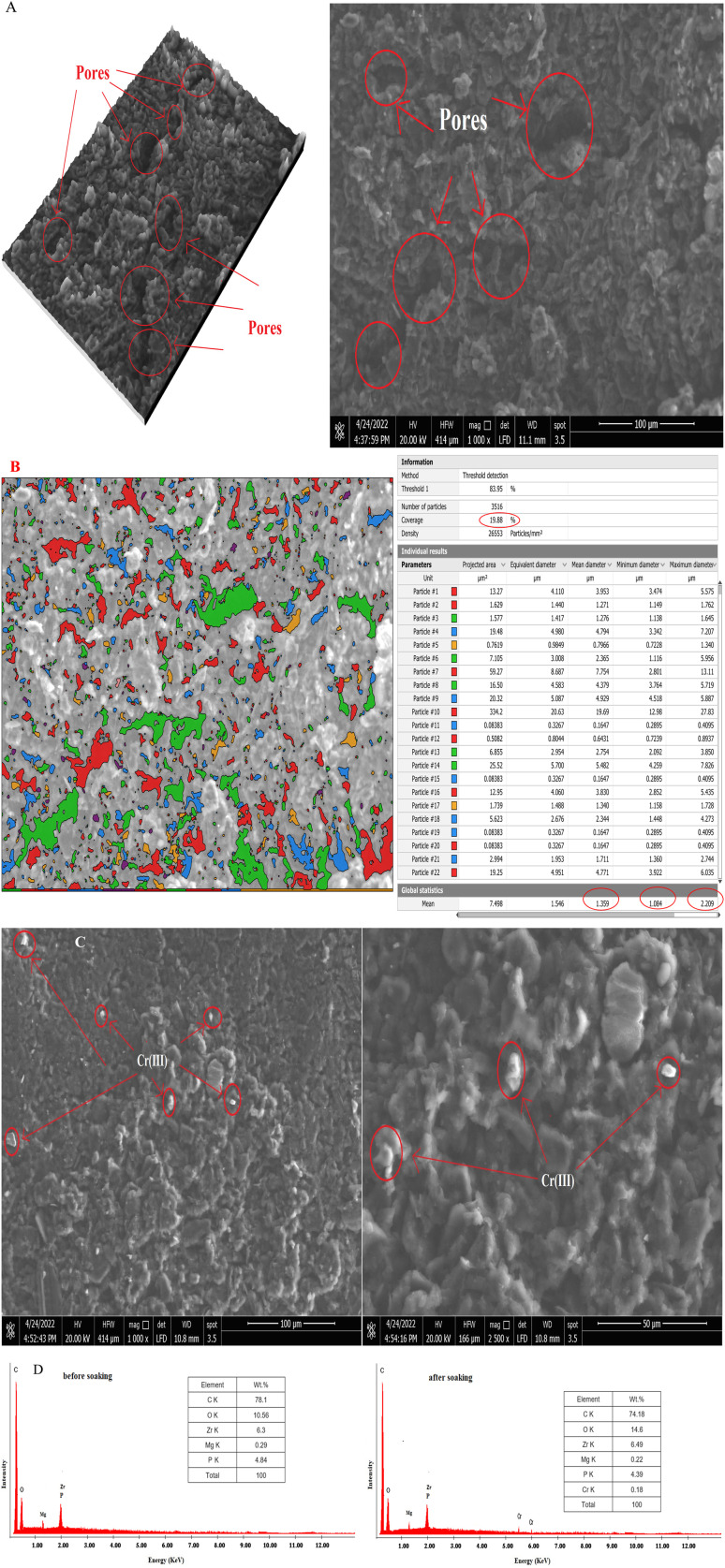
(A and B) SEM picture with pores, (C) SEM image after soaking, and (D) EDAX analysis. Surface SEM and EDX examination of the suggested sensor surface (10 mg nano-t-ZrO_2_ ionophore, 0.1 g TCP and 250 mg graphite).

In carbon paste electrodes, Fig. 4 (A and B), t-ZrO_2_ nanoparticles (ionophores) are coupled with graphite sheets and TCP as a solvent moderator to increase the pores area of CPE to 19.88% of total measured area, which is higher than the pores area of t-ZrO_2_ alone (13.54%), and to increase the average pore size to 1.359 nm with a range from 1.084 to 2.209 nm. Additionally, t-ZrO_2_ nanoparticles can form a connection with the Cr(iii)ion in real samples or pure solutions. This connection will alter the smoothness, roughen the electrode's surface, and cause the appearance of white spots as a consequence of the existence of Cr(iii) ions on the surface of the electrode following soaking, as evidenced by the SEM image (Fig. (4C)).

Additionally, the Cr(iii) ions existing in it and the quantitative data on the surface composition of the past both prior and following soaking in Cr(iii) ion solution had also reinforced this mechanism, as seen in Fig. (4D). This result enhanced the CPE's porosity and selectivity for the Cr(iii) ion. Therefore, it provides a capability for employing the suggested approach and the examined CPE for estimating the Cr(iii) ion in either pure solution or real samples.

### 3.7. Effect of plasticizer

Plasticizers are believed to hold a substantial impact on how CPEs behave. Among increased ionic mobility and sensing material solubility, they also have decreased bulk resistance of the electrode as a result of their polarity properties. They strengthen the mechanical connections between the various electroactive carbon particles to establish a consistent, compact combination.^4,15,16^ In accordance with the plasticization's lubricating hypothesis, the plasticizer molecules dispersed throughout the polymer and diminished the contacts (van der Waals forces). This enables the chains to pass each other speedily and leads to flexibility, softness, and elongation. It assumes that the plasticizer molecules are not permanently bound to the molecules but are free to self-associate. The non-polar section of the molecule must manage the polar section of the molecule so that it is not a powerful enough solvator to disrupt the crystallinity of materials. To offer softening, the molecule's polar part must be capable of binding reversibly. Plasticizers merely exhibit a strong affinity; they do not assist in a chemical process that results in bonding or grafting.

According to prior studies,^4,12^ the sensitivity and selectivity of ion-selective electrodes are known to be greatly influenced by the nature of the solvent mediator and any additives employed. Both the membrane's dielectric constant and the ionophore's mobility are influenced by the type of plasticizer. A plasticizer must have a variety of characteristics in order to be proper for use in sensors, according to Pérez *et al.*^49^. One or more of these features comprise a significant molecular weight, a low vapor pressure, and a large capacity to disintegrate the substrate and any additional additives existing in the matrix.

Five plasticizers with various polarities, including TCP, DBP, DHP, DOP, and *o*-NPOE plasticizers, were used to explore the impact of plasticizer type on the features of the researched sensor. It is realized that the electrode incorporating TCP frequently exhibits improved potentiometric responses, *i.e.*, sensitivity and linearity range of the calibration plots. Due to its significant dielectric constant and comparatively elevated molecular weight, it provides the best Nernstian slope (19.5 ± 0.10 mV decade^−1^) if compared with the alternative plasticizers, whose slopes values were 48.01 ± 1.2, 30.40 ± 1.2, 32.50 ± 0.1, and 32.70 ± 0.2 mV decade^−1^ for DBP, DHP, DOP, and *o*-NPOE plasticizers, respectively.

### 3.8. The pH effect

**Fig. 5:**
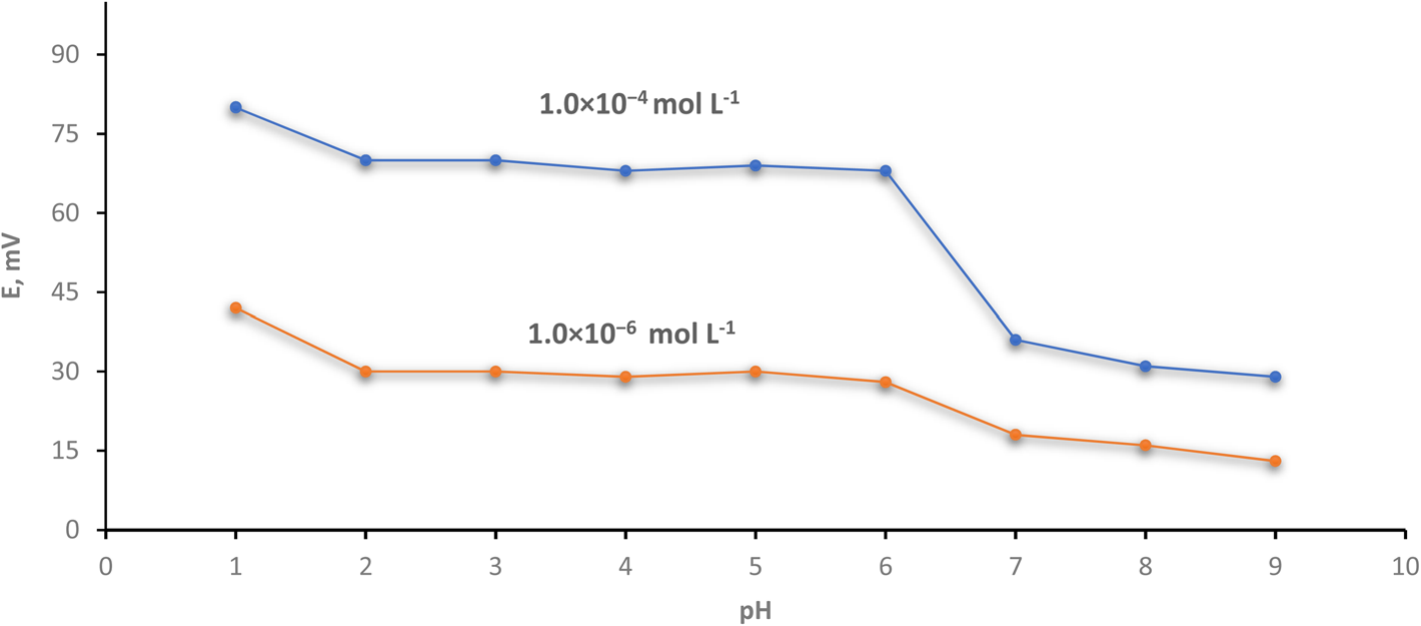
Validity of pH on the implementation of the investigated electrode.

By adding small volumes of HCl or/and NaOH (0.1–1 mol L^−1^ of each) and immersing the optimized electrode in 1.0 × 10^−4^ mol L^−1^ Cr(iii) solution, the influence of pH on the effectiveness of the investigated electrode was studied over the pH range of 1–9. Also, the resulting potential of 1.0 × 10^−6^ mol L^−1^ of Cr(iii) solution was recorded. Fig. (5) declares that the electrode has constant potential measurements (independent on the pH readings) in the pH range of 2.0–6.0. This means that the potential reading is still constant by changing pH. This interval contains wide potential stable range than Barati *et al.*^48^ which was 4–6.5 and the interval 4.5–7.7 is case of Abu-Shawish *et al.*^50^ Due to base precipitation (un protonated species) at high pH levels, the mV reading decreases and lower readings of EMF were obtained. Competitive protons at lower pH caused an increase in the potential values. In addition, at lower pH, partial protonation has an impact on the ionophore's capacity to bond Cr(iii) ions.^51^ To provide a potentiometric response, the polar functional groups can combine with metal ions to form complexes in competition with the ionophores.

According to Heidari and Masrournia,^52^ changes at elevated pH levels are caused by the development of soluble or insoluble chromium hydroxyl complexes, whereas changes at lower pH ranges are caused by protonation of the ionophore's donor atoms.

### 3.9. Lifetime

The lifetime of the paste of the proposed electrode was performed by calibration method periodical with standard solution of Cr(iii) and calculating the response slope (studying of time factor against Nernstian slope). From the ESI† Fig. (S1), the measurements were done every three days over 70 days, so they gave more accurate results than weekly measurements. Also, it explained that the lifetime of paste was 60 days with no changes in the Nernstian slope.

## 4. Selectivity coefficients

As shown in Table (1) and ESI† Fig. (S2), the effects of different inorganic cations and sugars on the performance of the electrodes were studied. The selectivity of an electrode is determined not only by the ion mobility, strength of association between the ion and the carrier in the electrode surface, variations in ionic size and permeabilities *etc.*, but also by the ionic strength and the ion concentration ratio factors. There are many methods which used for measuring the selectivity coefficients for the electrode (*K*_A,B_) as fixed interference method, separate solution method, matched potential method ….*etc.*

**Table 1:** Selectivity coefficient values for MCPE

Interfering compound	*K*_D,B_^pot^			
	SSM	FIM	FPM	MPM
Mn^2+^	9.62 × 10^−3^	2.20 × 10^−3^	4.20 × 10^−3^	—
Ni^2+^	7.74 × 10^−3^	4.42 × 10^−3^	7.72 × 10^−3^	—
Na^+^	1.09 × 10^−2^	0.51 × 10^−2^	0.38 × 10^−2^	—
Cu^2+^	1.93 × 10^−3^	2.23 × 10^−3^	1.95 × 10^−3^	—
Co^2+^	9.65 × 10^−4^	4.41 × 10^−3^	1.48 × 10^−3^	—
Ba^2+^	4.34 × 10^−3^	1.77 × 10^−3^	2.72 × 10^−3^	—
Pb^2+^	2.46 × 10^−2^	2.65 × 10^−2^	4.20 × 10^−2^	—
Ag^+^	6.87 × 10^−3^	7.11 × 10^−3^	8.03 × 10^−3^	—
Mg^2+^	5.93 × 10^−3^	6.56 × 10^−3^	4.66 × 10^−3^	—
Ca^2+^	4.65 × 10^−2^	8.09 × 10^−3^	9.71 × 10^−2^	—
Fe^3+^	7.03 × 10^−3^	9.05 × 10^−3^	5.77 × 10^−3^	—
Al^3+^	5.12 × 10^−3^	7.54 × 10^−3^	8.15 × 10^−3^	—
Zn^2+^	3.45 × 10^−3^	4.51 × 10^−3^	1.95 × 10^−3^	—
Ascorbic acid	1.96 × 10^−2^	—	—	—
Fructose	—	—	—	4.95 × 10^−2^
Maltose	—	—	—	1.39 × 10^−2^
Sucrose	—	—	—	4.34 × 10^−2^
Starch	—	—	—	1.09 × 10^−2^
Lactose	—	—	—	4.36 × 10^−3^

First, separate solution method (SSM) in which the potential of the primary ion and interfering ion is measured separately with respect to the activity of the interference (*a*_B_) and the primary ion (*a*_A_) is the same (*a*_A_ = *a*_B_).^2,12,16^ It depends on the Nicolskii–Eisenman equation.log *K*(_A,B_)^pot^ = ((*E*_2_ − *E*_1_)/*S*) + log (*a*_A_/(*a*_B_)^(Z_A_/Z_B_)^)where *E*_1_ is the potential measure of Cr(iii) solution and *E*_2_ is the potential of the interfering compound (B). *Z*_1_ and *Z*_2_ are the charges of Cr(iii) and interfering species (B) and *S* is the slope of the electrode calibration graph.

Second, fixed interfering method (FIM) involved a fixed constant activity of the interfering ion *a*_B_, and titration with varying activity of the primary ion. The selectivity coefficient is calculated from the following equation:^2,53^*K*_AB_ = (*a*_A_/(*a*_B_)^Z_A_/Z_B_^)where *a*_A_ is the activity of the primary ion (Cr(iii)) at the lower detection limit in the presence of interfering ion and *Z*_A_ and *Z*_B_ are the charge of ions (*Z*_A_ not equal *Z*_B_). The values of potential were plotted against the concentration of interfering ion.

Third, in fixed primary method (FPM), the values of potential were plotted against the concentration of the interfering ion where the activity of the primary ion is constant.^54^ Finally, matched potential method (MPM) is unique because it doesn't depend on the Nicolskii–Eisenman equation like the other methods. Also, it neglects the effect of charges and the Nernstian response of both the primary and interfering ions. So, it is suitable for neutral species and sugars like lactose, starch and fructose and not suitable for charged species like Fe(iii), Na(i), Pb(ii) and others. Here, the potentiometric selectivity coefficient *K*_A,B_ is defined as the activity ratio of primary and interfering ions that give the same potential change under identical conditions. At first, a known activity (*a*_A’_) of the primary ion solution is added into a reference solution that contains a fixed activity (*a*_A_) of primary ions, and the corresponding potential change (Δ*E*) is recorded. Next, a solution of an interfering ion is added to the reference solution until the same potential change (Δ*E*) is recorded. The change in potential produced at the constant background of the primary ion must be the same in both cases.^2,18^*K*_A,B_ = (*a*_A’_ − *a*_A_)/*a*_B_

From Table (1), it is clear that high selectivity coefficient values in the state of sugars, ascorbic acid and other metal ions is observed. This is due to the polarity, atomic size, and lipophilic nature of their molecules in respect to Cr(iii) solution. Also, it explains usage of diverse metal ions with varying atomic sizes and oxidation values were employed as interfering entities. It is noted that values of selectivity coefficient are smaller than 1.0 indicating that the sensor is responding more to primary ion than to interfering ions and in such cases the sensor is said to be more selective to primary ion over interfering ions.^2,12,16,52^

### 4.1. Effect of temperature

The temperature's impact on the potentiometric electrode's performance was assessed from 10 to 60 °C, and the isothermal coefficient (d*E*°/d*T*) will be adjusted for each electrode in accordance with the giving equation:^19^
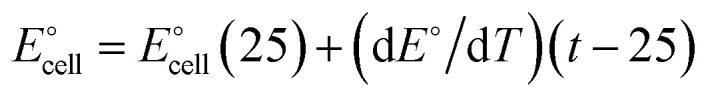
.

In the temperature range of 10 to 60 °C, it is noticeable that the electrode provided a satisfactory Nernstian reaction. The isothermal coefficient is expressed by the slope of the resulting straight line, which was established to be 0.743 × 10^−3^ V per °C. The extraordinarily high thermal stability of the electrode was evidenced by the extremely minimal value of the isothermal coefficient. This shows that the tested electrode may be employed up to 60 °C without significantly deviating from Nernstian behavior with reliable results for the measurement of Cr(iii) ions.^16^

### 4.2. Effect of response time

**Fig. 6:**
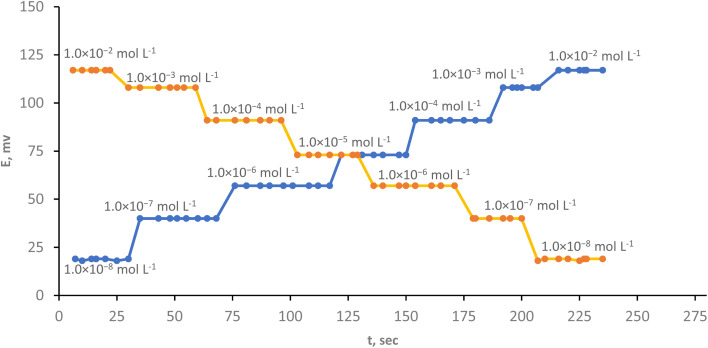
Effect of response time on the electrode performance.

The phrase “response time” refers to the average duration of time it takes for the electrode to balance at a voltage that is within ±1 mV of the final equilibrium value. The time constant of the electrode's response function is substantially bigger, this due to several factors, such as the measurement device's time constant, the rate of ion transfer reaction at the paste–pattern interface, the impedance of the paste's equivalent electric circuit, or the stability of a liquid-junction potential at the reference electrode, directly impact the entire response time.^53^Fig. (6) makes it evident that the electrode has a quick response time of 7 s even when it measured forward (from lower concentration to higher concentration) or backward (from higher concentration to lower concentration). This is owing to the inclusion of the optimum ion pair content and a good solvent moderator. Contrasting to our finding, Shojaei *et al.*^55^ reported that the sensor's reaction time for identifying Cr(iii) ions is around 10 s and Barati, *et al.* response sensor's time was smaller than 15 s.^48^

### 4.3. Method validation

Considering the ideal experimental circumstances, the analytical approach was established in accordance with the standards of the International Conference for Harmonization (ICH): accuracy, specificity, precision, limit of detection (LOD), linearity and limit of quantification (LOQ) were realized for standard Cr(iii) ion solution.^7,56^ LOD stands for lowest detectable dose of the examined substance in the sample, although it might not be evaluated with a tolerable level of uncertainty. When the concentration varies slightly without causing any discernible change in the response, it was the concentration of detected ions at the junction of the extrapolated linear portion of the calibration curve reflecting the normal slope of the electrode and the horizontal line denoting the voltage when the concentration is so low that small change in concentration don't produce any detectable change in the response of electrode.^4^ The LOD was 1.0 × 10^−8^ mol L^−1^, which is better than the value published in Barati *et al.*^48^ which was 3.1 × 10^−7^ mol L^−1^ in the concentration from 5.0 × 10^−7^ to 1.0 × 10^−3^ mol L^−1^. The data revealed that the sensor has a high sensitivity and can be used to detect low levels of Cr(iii) ion concentration, as revealed in ESI† Table (S2).

LOQ is The smallest amount of a material that may be precisely and correctly quantified in a sample matrix.^12^ According to ESI† Table (S2) and Fig. (S3), it was discovered that the LOQ was 3.33 × 10^−8^ mol L^−1^, it is indicated that this electrode has more sensitive measurements towards the determination of Cr(iii) (LOQ = 10 LOD/3).

Accuracy is a crucial requirement for analytical techniques and refers to the extent to which the resulting value is close to the true or considered as the reference value. Table 2 shows that the high percentage recovery statistics ensure the method's high accuracy. The extent to which outcomes agree with one another is a measure of precision. The replicated analyses' standard or relative standard deviations are generally employed to express it. Using pure Cr(iii) solution and some real samples, inter- and intra-day precisions were considered across five replicates. The minimal relative standard deviation values reflect the method's outstanding repeatability and reproducibility (Table (2)).

**Table 2:** Evaluation of examined CPE's intra- and inter-day accuracy and precision; for *n* = 5

Form	[Cr(iii)]	Intra-day			Inter-day		
Type of sample	Taken mg mL^−1^	Found mg mL^−1^	Recovery (%)±SD	RSD%	Found mg mL^−1^	Recovery (%)±SD	RSD%
Pure	0.239	0.233	97.49 ± 0.008	3.43	0.233	97.49 ± 0.007	3.00
Water	0.239	0.235	98.33 ± 0.007	2.30	0.234	97.90 ± 0.009	3.85
Tea	0.239	0.240	100.4 ± 0.009	3.75	0.229	95.82 ± 0.012	3.34
Cheese	0.239	0.241	100.8 ± 0.002	0.83	0.237	99.16 ± 0.004	1.69
Milk	0.239	0.238	99.58 ± 0.003	1.26	0.238	99.58 ± 0.003	1.26
Honey	0.239	0.240	100.4 ± 0.009	3.75	0.237	99.16 ± 0.001	0.38
Coffee	0.239	0.237	99.16 ± 0.001	0.42	0.236	98.74 ± 0.004	1.69
Cosmetic	0.239	0.238	99.58 ± 0.002	0.84	0.235	98.33 ± 0.002	0.85

The degree to which the calibration plot of electrochemical potential *vs.* concentration resembles a straight line is known as linearity. Using a standard Cr(iii) solution, the standard calibration curve was generated. As indicated in ESI †Table (S2) and Fig. (S3), a linear relation between negative log[Cr(iii)] and potential (mV) was established.^57,58^ The linear range for the investigated CPE was 1.0 × 10^−8^ – 1.0 × 10^−2^ mol L^−1^.

The process of determining specificity involved screening for any interference from typical excipients. The outcome of the suggested method was found to be unaffected by these components, ESI† Fig. (S2).

### 4.4. Applications

Utilizing the potentiometric calibration approach, the designed sensor was employed to recognize the chromic ion in a variety of samples, including tea, coffee, milk, cheese, honey, and cosmetics. Inductively coupled plasma mass spectrometry (ICP-MS) findings and potentiometric calibration method results were compared,^19–21^ and the results of this comparison were presented in Table (3). In regards of recovery and relative standard deviation, the analysis of the Cr(iii) ion in both pure solution and spiked samples achieved encourageable findings, indicating the superior accuracy and precision of the suggested electrode. These results also compared with results of ICP technique. It is found that the values of F-test ranged from 0.01 to 4.80. On the other hand, the values of *t*-test were ranged from 0.02 to 2.40. According to this comparison, it showed no noticeable changes between the two approaches, hence highlighting its benefits. It is clear from these data that the calculated *F*- and *t*-test values are lower than the tabulated values at 95% confidence level supporting the nonsignificant difference between the reported potentiometric method and ICP method.

**Table 3:** Comparison of ICP-MS and studied CPE for the detection of pure Cr(iii) and other spiked authentic samples^a^

Type of method	Type of Sample	[Cr(iii)]			
		Taken mg mL^−1^	Found mg mL^−1^	Recovery (%)±SD	RSD%
Potentiometry	Pure^b^	0.0260	0.0259	99.62 ± 0.0004	1.54
	Water^c^		0.0246	94.62 ± 0.0008	3.25
	Tea		0.0256	98.46 ± 0.0006	2.69
	Cheese		0.0251	96.54 ± 0.0005	2.31
	Milk		0.0248	95.83 ± 0.0009	3.77
	Honey		0.0257	98.85 ± 0.0010	4.14
	Coffee		0.0259	99.62 ± 0.0009	3.43
	Cosmetic		0.0258	99.23 ± 0.0009	3.56
ICP-MS	Pure^b^	0.0260	0.0245	94.23 ± 0.0006	2.44
	Water^c^		0.0249	95.77 ± 0.0011	4.01
	Tea		0.0255	98.08 ± 0.0003	1.18
	Cheese		0.0254	97.69 ± 0.0093	3.54
	Milk		0.0255	98.08 ± 0.0002	1.96
	Honey		0.0248	95.38 ± 0.0012	4.03
	Coffee		0.0251	96.50 ± 0.0007	2.78
	Cosmetic		0.0257	98.85 ± 0.0004	1.56

a At *n* = 5, 95% confidence level, *F*-test (tabulated) = 5.190, *t*-test (tabulated) = 2.571 *F*-test (experimental) = 0.01–4.80, *t*-test (experimental) = 0.02–2.40.

b Pure: Cr(iii) solution in double distilled water.

c Water: tap water spiked with known amount of Cr(iii) ion.

### 4.5. Comparative study

It would be acceptable to state that this sensor displayed increased selectivity and sensitivity for the selective detection of Cr(iii) ions in comparison to the latterly described potentiometric sensors^2,50,59–64^ (Table (4)). This could be explained by the exceptional characteristics of the used nano t-ZrO_2_ ionophore, such as its surface area, porous structure, mechanical, and conductivity capabilities. It was clear from Table (4) that the proposed sensor has LOD value, linear concentration range and pH range comparable or sometimes better than the reported ones. The potentiometry approach additionally offers advantages including simplicity, low cost, the lack of sample fabrication, and quick response. CPEs are reliable, chemically inert, affordable, renewable, and stable.

**Table 4:** Comparison between the previously reported sensors and proposed sensor for Cr(iii) ion determination

Modifier	Slope (mV decade^−1^)	Detection limit, mol L^−1^	Linear range, mol L^−1^	pH range	Response time, s	Lifetime (month)	Interfering ions with log *k*_Cr(III),*j*_^pot^ ≥ −2	Ref.
*N*-(Aceto acetanilide)-1,2-diaminoethane	19.8	5.60 × 10^−8^	8.9 × 10^−8^–1.0 × 10^−1^	2.0–7.0	10	3	No interference	62
*N*, *N* bis(salicylidene)-*o*-phenylenediaminatechromium(iii)	20.1	1.80 × 10^−6^	7.5 × 10^−6^–1.0 × 10^−2^	4.5–7.7	8	Not mentioned	Pb(ii), Hg(ii) and Al(iii)	50
5-Amino-1-phenyl-1H-pyrazole-4-carboxamide	19.6	5.30 × 10^−7^	1.0 × 10^−6^–1.0 × 10^−1^	3.2–6.3	10	2	Ca(ii)	61
1-[(2-Hydroxy ethyl) amino]-4-methyl-9H-thioxanthen-9-one	20.51	1.60 × 10^−7^	3.2 × 10^−7^–1.0 × 10^−1^	4.8–6.3	10	Not mentioned	Co(ii), Sr(ii), Mg(ii) Ba(ii) and K(i)	60
*p*-(4-Acetanilidazo) calix^4^ arene	19.8	Not mentioned	9.8 × 10^−7^–1.0 × 10^−1^	2.8–5.7	10	3	Fe(iii)	63
1-(2-(1H-Imidazole-1-yl)-1-(4-methoxyphenyl) ethylidene)-2-phenyl hydrazine	19.6	6.80 × 10^−8^	8.4 × 10^−8^–1.0 × 10^−2^	3.3–5.9	10	2	Al(iii) and Ag(i)	59
*N*-[4-(Dimethylamino)benzylidene]-6-nitro-1,3-benzothiazol-2-amine	20	2.0 × 10^−7^	4.0 × 10^−6^–1.0 × 10^−1^	2.8–5.1	15	5	No interference	2
Nano chromium complex	18.8	1.00 × 10^−8^	1.0 × 10^−8^–1.0 × 10^−2^	2.0–6.0	8	3	Fe(iii)	64
t-ZrO_2_ nanoparticles	19.5	1.00 × 10^−8^	1.0 × 10^−8^–1.0 × 10^−2^	2.0–6.0	8	2	No interference	This study

## 5. Conclusion

Pure nano-t-ZrO_2_ was synthesized utilizing a modified co-precipitation method with a 14% MgO stabilizer, and it was then fully characterized *via* different characterization methods. The selectivity and sensitivity toward the Cr(iii) ion improved after the carbon paste composition was properly optimized. The redesigned electrode had favorable Nernstian slope, a low detection limit, a high recovery percentage, low standard deviation and relative standard deviation values. This straightforward, affordable, and renewable chromic ion sensor might be used in various authentic samples. In light of this, the newly developed sensor might be a useful addition to the list of Cr(iii) sensors already in use.

## Conflicts of interest

The authors affirm that they do not have any conflicting interests.

## Supplementary Material

RA-013-D3RA01563G-s001
