# Supplementary material for: Modified carbon paste ion selective electrode for determining Cr(iii) ions in aqueous solutions and some real samples using tetragonal zirconia nanoparticles
Source: RSC Adv. 2023 Apr 11;13(16):11201–14. doi: 10.1039/d3ra01563g (PMC10086672; doi:10.1039/d3ra01563g)
Supplement: RA-013-D3RA01563G-s001 [file RA-013-D3RA01563G-s001.pdf]

Electronic Supplementary Material (ESI) for RSC Advances.
This journal is © The Royal Society of Chemistry 2023

Synthesis and characterization of tetragonal zirconia nanoparticles for use in a carbon paste ion selective electrode to determine Cr(III) ions in aqueous solutions and some real samples

Omar A. Fouad[1*], Mohamed M.S. Wahsh[2], Gehad G. Mohamed[1,3], Maher MI El Dessouky[1], Maysa R. Mostafa[1]

[1] Chemistry Department, Faculty of Science, Cairo University, 12613 Giza, Egypt

[2] Refractories, Ceramics and Building Materials Department, National Research Centre, 12622, Cairo, Egypt

[3] Nanoscience Department, Basic and Applied Sciences Institute, Egypt-Japan University of Science and Technology, New Borg El Arab, Alexandria, 21934, Egypt

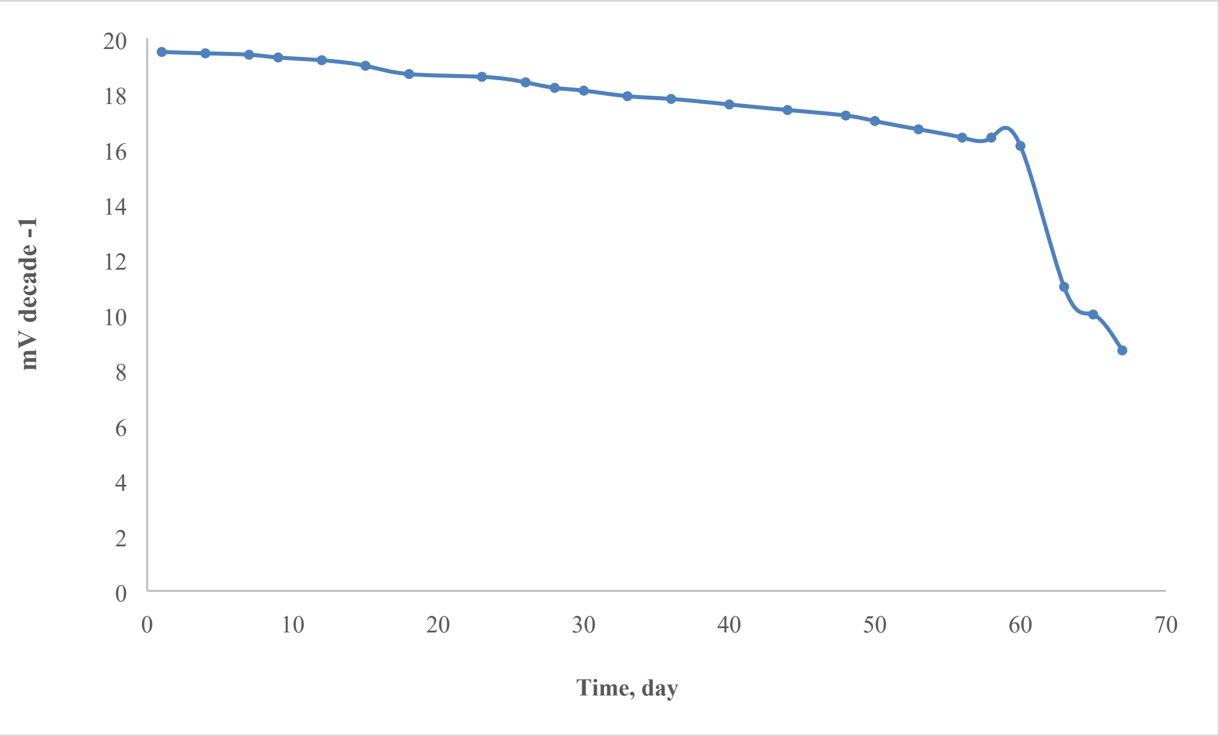

Supplementary Figure S1. Effect of lifetime of the paste on the performance of electrode.

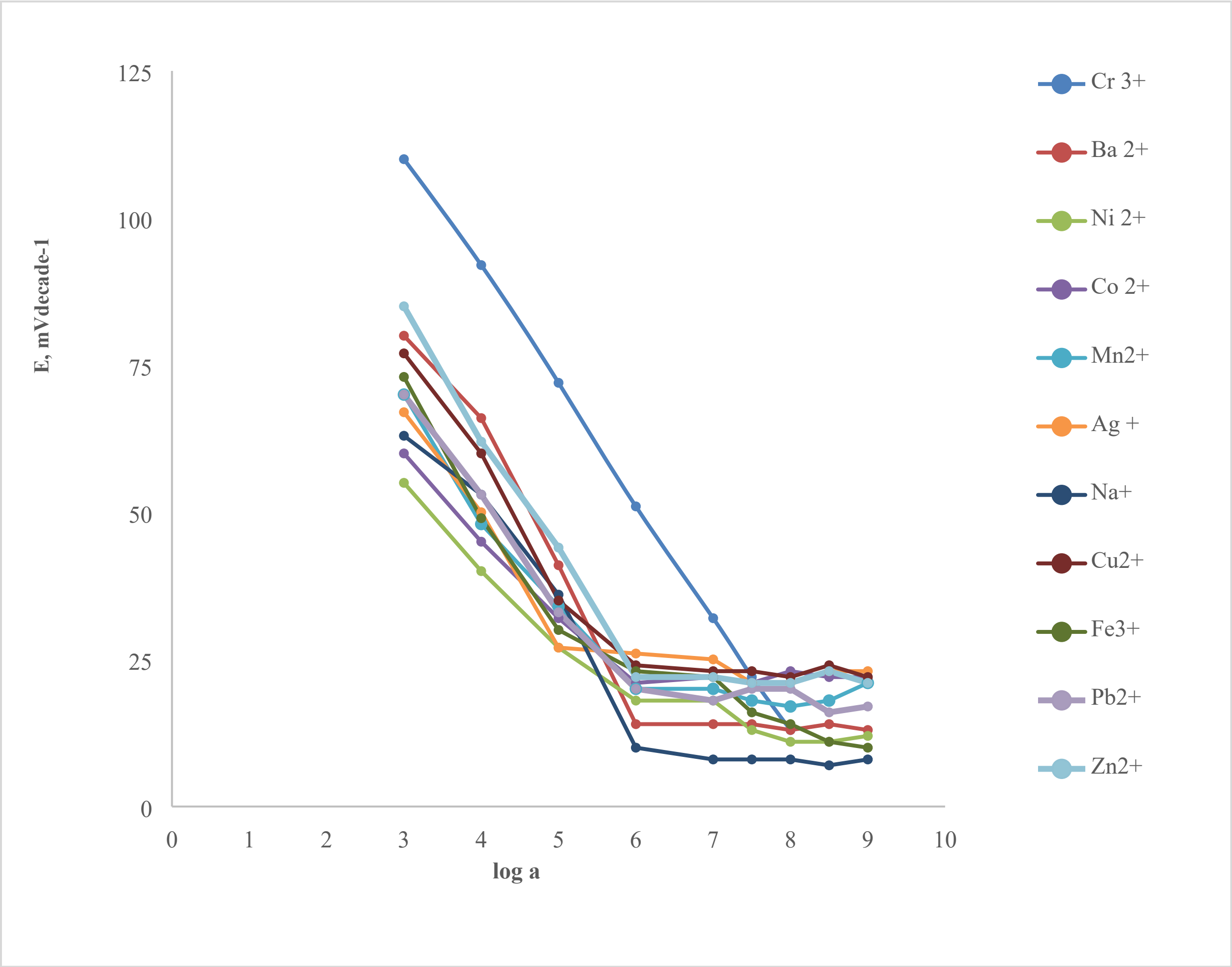

Supplementary Figure S2. The potential response of the proposed sensor for diverse metal ions.

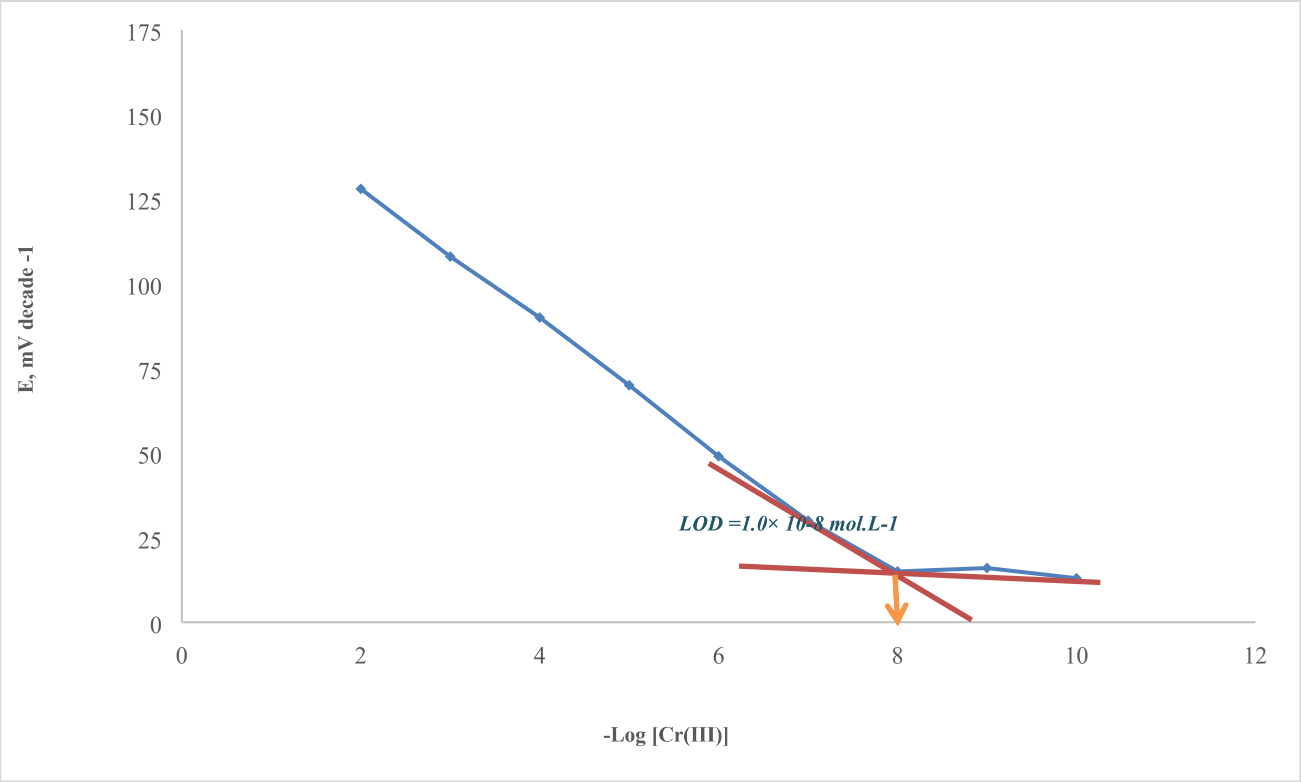

Supplementary Figure S3. The calibration curve of the proposed sensor.

Supplementary Table S1. Effect of content of t-ZrO_2 and plasticizer on the performance of carbon past electrode.

Graphite (mg)	ZrO_2 (mg)	MgO (%)	Name of plasticizer	Plasticizer (g)	Slope(mV decade^{-1})	R^2	Linear range
250	5	14	TCP	0.117	41.70±0.19	0.886	1.0×10^{-5}- 1.0×10^{-3}
250	10	14	TCP	0.117	19.50±0.10	0.998	1.0×10^{-8}-1.0×10^{-2}
250	15	14	TCP	0.117	30.00±0.50	0.882	1.0×10^{-5}-1.0×10^{-3}
250	10	14	DOP	0.099	32.50±0.10	0.890	1.0×10^{-5}-1.0×10^{-3}
250	10	14	NOP	0.104	32.70±0.20	0.850	1.0×10^{-5}-1.0×10^{-3}
250	10	14	DBP	0.104	48.01±1.20	0.900	1.0×10^{-5}-1.0×10^{-3}
250	10	14	DHP	0.101	30.40±1.20	0.811	1.0×10^{-5}-1.0×10^{-3}

Supplementary Table S2. Summary of factors that affect the performance of the proposed electrode.

Parameters	Value
Best content	10 mg ZrO_2 14 %
Slope	19.50 ± 0.10 mVdecade^{-1}
Plasticizer	TCP
Effect of Response time /sec.	7
Effect of temperature V/C	0.743 ×10^{-3} V/C
Effect of pH	2.0-6.0
Effect of lifetime / Days	60
LOD	1.0×10^{-8} mol L^{-1}
LOQ	3.33×10^{-8} mol L^{-1}
Linearity	1.0×10^{-8} - 1.0×10^{-2} mol L^{-1}
